# Decreased expression levels of complement regulator CD55 contribute to the development of bullous pemphigoid

**DOI:** 10.18632/oncotarget.21216

**Published:** 2017-09-23

**Authors:** Pei Qiao, Er-Le Dang, Hui Fang, Jie-Yu Zhang, Bing Li, Sheng-Xian Shen, Yi-Xin Luo, Jie Lei, Shuai Shao, Hong-Jiang Qiao, Gang Wang

**Affiliations:** ^1^ Department of Dermatology, Xijing Hospital, Fourth Military Medical University, Xi’an, Shaanxi Province, China

**Keywords:** bullous pemphigoid, complement, CD55, ERK1/2, autoimmunity

## Abstract

Bullous pemphigoid is a common autoimmune blistering disease of the elderly associated with autoantibody-mediated complement activation, and complement dysregulation is critical for its pathogenesis. As a crucial regulator of the complement system, CD55 has been widely studied in autoimmune diseases. Here, we investigated the involvement of CD55 in bullous pemphigoid, as little is known regarding its role in this disease. We found that CD55 levels were significantly lower in the lesions of patients with bullous pemphigoid (n = 8) compared to those in skin samples from healthy controls (n = 6). Interestingly, CD55 depletion in HaCaT human keratinocytes enhanced autoantibody-mediated complement activation. Moreover, complement activation was blocked by exogenous recombinant CD55 protein in both skin sections and keratinocytes exposed to pathogenic antibodies from patients with bullous pemphigoid. Notably, a significant increase in the expression of TNF-α and IFN-γ, administration of which downregulated CD55 levels in HaCaT cells, was observed in the sera of patients with bullous pemphigoid (n = 38) compared to that in healthy controls (n = 19). We found that ERK1/2 is involved in both TNF-α- and IFN-γ-induced CD55 downregulation. Thus, CD55 deficiency is a crucial factor in bullous pemphigoid pathogenesis, suggesting that increasing CD55 levels may exert a therapeutic effect.

## INTRODUCTION

Bullous pemphigoid is the most common autoimmune subepidermal blistering disease and is characterized by the production of autoantibodies that directly target the bullous pemphigoid antigen 180 (BP180 or COL17A1) within the dermoepidermal junction (DEJ) [1, 2]. Targeting of BP180 by autoantibodies leads to activation of the complement system, which mediates a series of inflammatory events, including dermal mast cell degranulation, generation of eosinophil-rich infiltrates, and subsequent blister formation [3, 4]. Autoantibodies and the key complement component C3b can often be detected at the basement membrane zone (BMZ) of the lesional skin of bullous pemphigoid patients [5]. In addition, it has been reported that complement component C5-deficient mice do not develop bullous pemphigoid following exposure to the pathogenic anti-BP180 IgG, which is used to induce this disease model. Similarly, mice injected with F(ab′)_2_ fragments of the pathogenic IgG fail to develop this condition [6, 7]. Complement activation is widely recognized as an important step in the pathogenesis of bullous pemphigoid; however, little is known regarding the pathogenic importance of upstream regulators of this process.

The complement system plays a key role in the immune response and can be activated through three routes: the classical, lectin, and alternative pathway [8–10]. All three result in production of the same final effector molecules as part of the complement cascade, including C3b, C3a, and the terminal membrane attack complex (MAC), which act as inflammatory mediators in humoral and cellular immunity [11]. Under normal conditions, cells resist inappropriate complement attack by expression of membrane-bound complement regulatory proteins, including CD35 (CR1), CD46, CD55, and CD59, which prevent activation of the complement system and provide essential protection against self-damage [9, 11, 12]. It has been reported that dysregulation of these proteins directly affects the progression of several autoimmune diseases, including systemic lupus erythematosus (SLE) and rheumatoid arthritis [13–15].

CD55, a 70-kDa globular glycoprotein, is anchored to the cell membrane by glycosylphosphatidylinositol and possesses four short consensus repeat domains. Classically, CD55 regulates activation of the immune system by inhibiting the formation of new C3 and C5 convertases and accelerating the degradation of those already formed, protecting cells from complement activation-induced damage [16, 17]. In some autoimmune diseases, CD55 expression and function are impaired. For instance, Richaud-Patin et al. reported the diminished expression of CD55 and CD59 on the red blood cells of patients with SLE and having secondary autoimmune hemolytic anemia (AIHA) [18]. In addition, CD55 expression has been shown to be negatively correlated with disease activity and complement system activation in SLE patients with neutropenia [19, 20]. Jindrich et al. demonstrated that CD55 deficiency significantly affects adaptive immune responses and worsens experimental autoimmune myasthenia gravis outcome and is associated with increased levels of serum cytokines [21, 22].

We therefore hypothesized that loss of CD55, a typical complement regulator acting via suppression of C3 and C5 activity, contributes to bullous pemphigoid development. To test this hypothesis, we assessed CD55 expression in the lesions of bullous pemphigoid patients and examined the mechanism *in vitro*. Our results demonstrate a role for CD55 in bullous pemphigoid progression and suggest that it may serve as a therapeutic target for this disease.

## RESULTS

### CD55 expression is decreased in epidermal keratinocytes from patients with bullous pemphigoid

**Figure 1:**
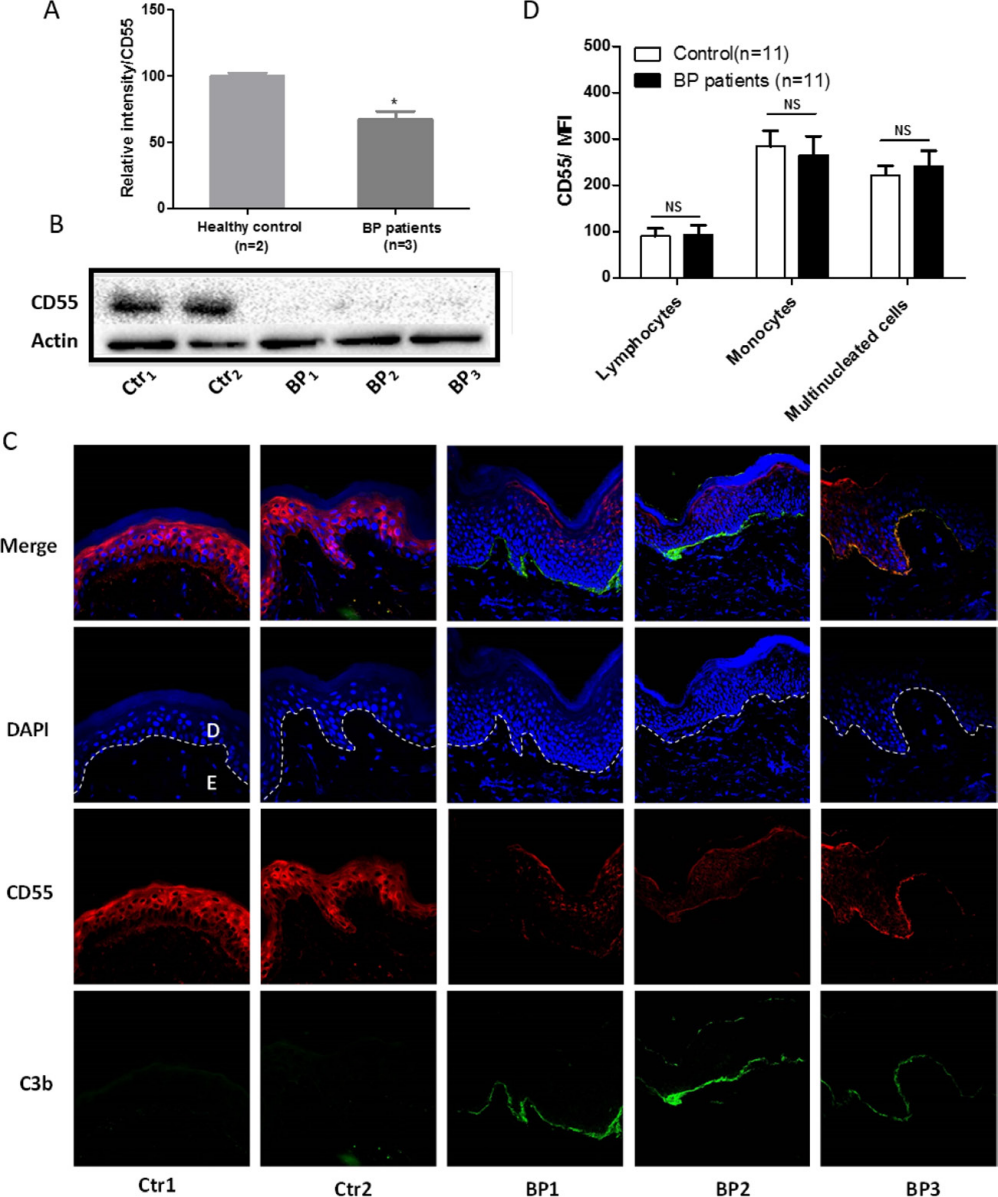
CD55 downregulation in the lesional skin of patients with bullous pemphigoid CD55 expression in bullous pemphigoid skin sections was examined by qRT-PCR **(A)** and western blotting **(B)**. **(C)** Normal skin sections and lesional skin specimens from patients with bullous pemphigoid were subjected to immunofluorescent staining to measure CD55 expression in epidermal keratinocytes. **(D)** Flow cytometric analysis of CD55 expression on peripheral blood cells. Results are presented as the means ± SEM from 11 independent observations. D, dermal; E, epidermal. NS, no significant difference between controls and bullous pemphigoid patients. DAPI-stained nuclei can be seen in blue. Scale bar, 100 nm.

qRT-PCR and Western blotting revealed that CD55 was downregulated in skin specimens from three patients with bullous pemphigoid in comparison to those from two healthy controls (Figure 1A-1B). To verify this result, we performed immunofluorescent staining of tissue sample paraffin sections from 8 patients with bullous pemphigoid. In 6 healthy controls, CD55 was present at high levels in the stratum spinosum and stratum granulosum of the epidermis and was moderately expressed in the basal membrane. In contrast, we observed only weak staining of CD55 in epidermal specimens from patients with bullous pemphigoid (Figure 1C), consistent with the results of our western blot analysis. Together, these results indicate that CD55 expression is abnormally downregulated in the epidermis of patients with bullous pemphigoid, and this may be responsible for the activation of the complement system and C3b deposition in the DEJ of BP patients. In addition, we examined CD55 expression in peripheral blood cells from patients with bullous pemphigoid by flow cytometry. Compared with that in healthy controls, expression of CD55 in peripheral blood cells, including lymphocytes, monocytes, and multinucleated cells, did not differ (Figure 1D).

### Pathogenic IgG-mediated complement activation in bullous pemphigoid is enhanced by CD55 depletion *in vitro*

**Figure 2:**
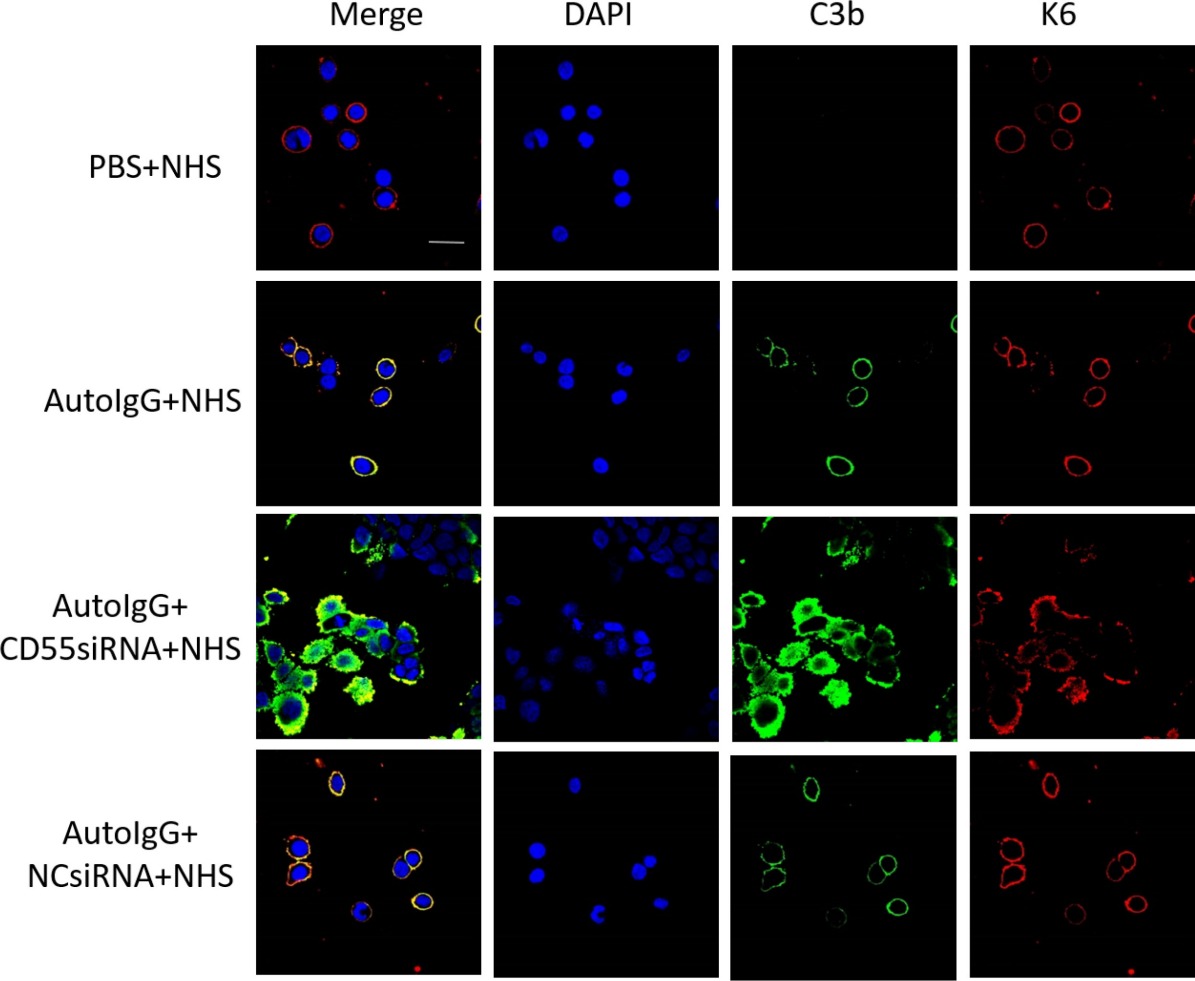
CD55 depletion enhances auto-IgG-mediated C3b deposition in HaCaT cells HaCaT cells transfected with CD55 siRNA were incubated with purified auto-IgG overnight at 37°C. Fresh serum from healthy controls containing complement components was then added to mimic bullous pemphigoid pathogenesis. Keratin 6 (red) and C3b (green) deposition was detected by immunofluorescent staining to determine the extent of complement activation. Results are representative of three independent experiments. Scale bar, 50 nm. Auto-IgG, pathogenic IgG from the sera of patients with bullous pemphigoid; NHS, normal human serum; NC siRNA, negative control siRNA.

Based on the above findings, we next investigated whether abnormal expression of CD55 is involved in the pathogenesis of bullous pemphigoid. HaCaT human keratinocytes were treated with pathogenic IgG from patients with this disease and complement components from the sera of healthy controls. The keratinocyte marker Keratin 6 and C3b deposition was then evaluated 2 h later by immunofluorescent staining. In addition, CD55 expression was reduced by short interfering RNA (siRNA) transfection, the effectiveness of which is shown in Supplementary Figure 1. As indicated in Figure 2, C3b deposition was clearly observed on HaCaT cell membranes after incubation with pathogenic autoantibodies and complement components. In contrast, no signal was observed in the negative control treated with PBS. siRNA-mediated CD55 downregulation resulted in a significant increase in C3b staining compared to that of both the un-transfected positive and NC siRNA negative control (Figure 2). Thus, CD55 deficiency leads to increased autoantibody-mediated C3b deposition in keratinocytes.

### Recombinant CD55 protein inhibits bullous pemphigoid IgG-mediated C3b deposition

**Figure 3:**
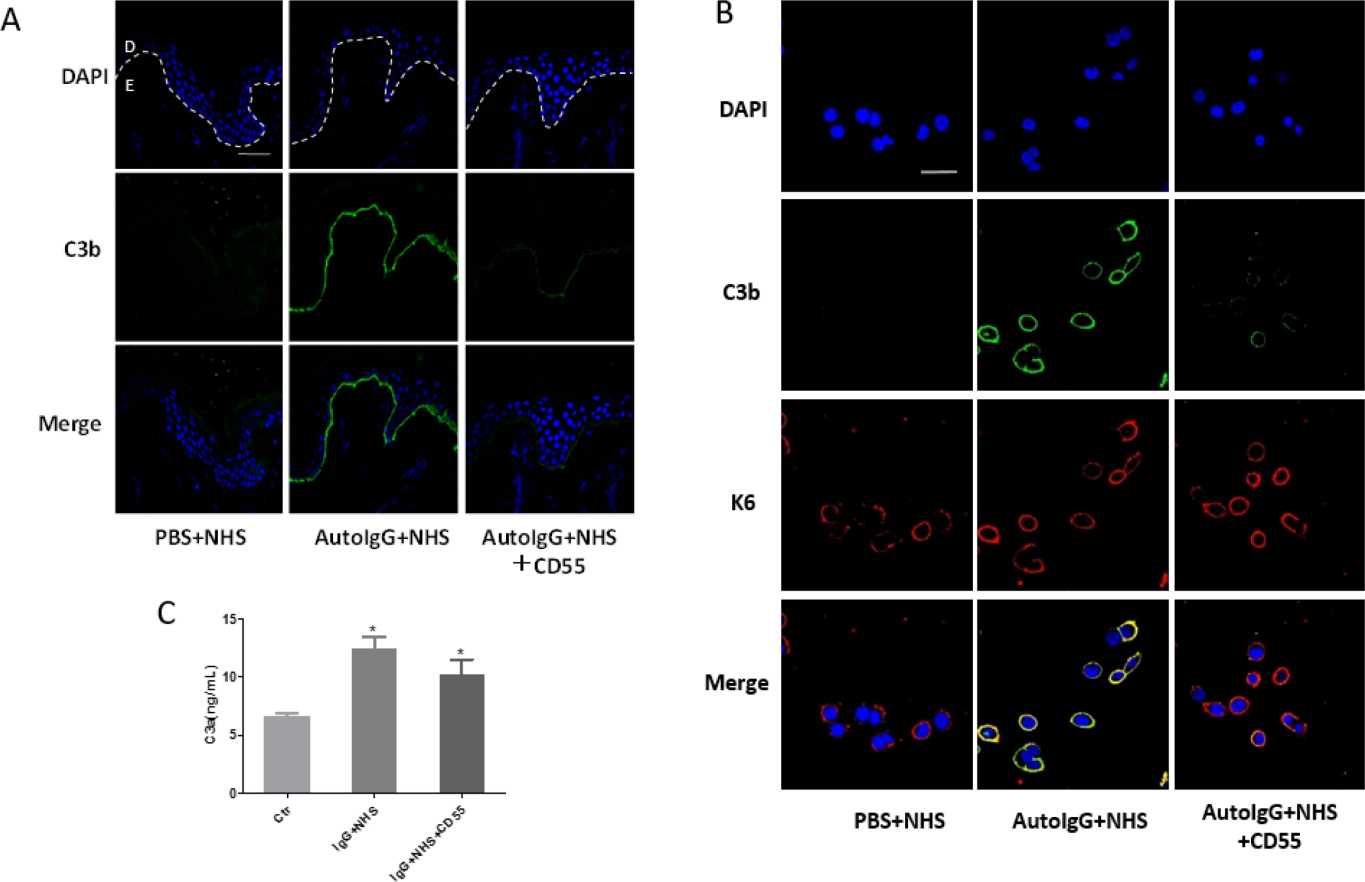
Recombinant CD55 inhibits autoantibody-mediated C3b deposition in bullous pemphigoid pathogenesis **(A)** Frozen skin sections from healthy controls and **(B)** HaCaT cells were incubated with purified auto-IgG and fresh serum from healthy controls containing complement components. C3b deposition was detected by immunofluorescent staining to determine the complement activation level. **(C)** The C3a expression in the supernatant of HaCaT cells was detected by ELISA. Results are representative of three independent experiments. Scale bars, 100 nm in **(A)** and 50 nm in **(B)**. Auto-IgG, pathogenic IgG from the sera of bullous pemphigoid patients; NHS, normal human serum. D, dermal; E, epidermal.

To further investigate the regulatory role of CD55 in bullous pemphigoid pathogenesis, we next exposed frozen skin sections from healthy volunteers and HaCaT cells to pathogenic IgG and fresh serum from healthy controls containing complement components. Immunofluorescent staining was then performed 2 h later to evaluate C3b deposition. As expected, clear deposition was noted both in HaCaT cells and in the BMZ of healthy skin samples incubated with pathogenic IgG. Notably, this effect was inhibited by treatment with 10 μg/ml recombinant human CD55 protein (Figure 3A-3B). In addition, the expression of C3a in the supernatant was inhibited by CD55 treatment in HaCaT cells, which is consistent with the results of C3b (Figure 3C). Taken together, these results suggest that lack of CD55 leads to complement activation in the pathogenesis of bullous pemphigoid.

### Elevated TNF-α and IFN-γ levels are present in the sera of patients with bullous pemphigoid and affect CD55 expression in HaCaT cells

**Figure 4:**
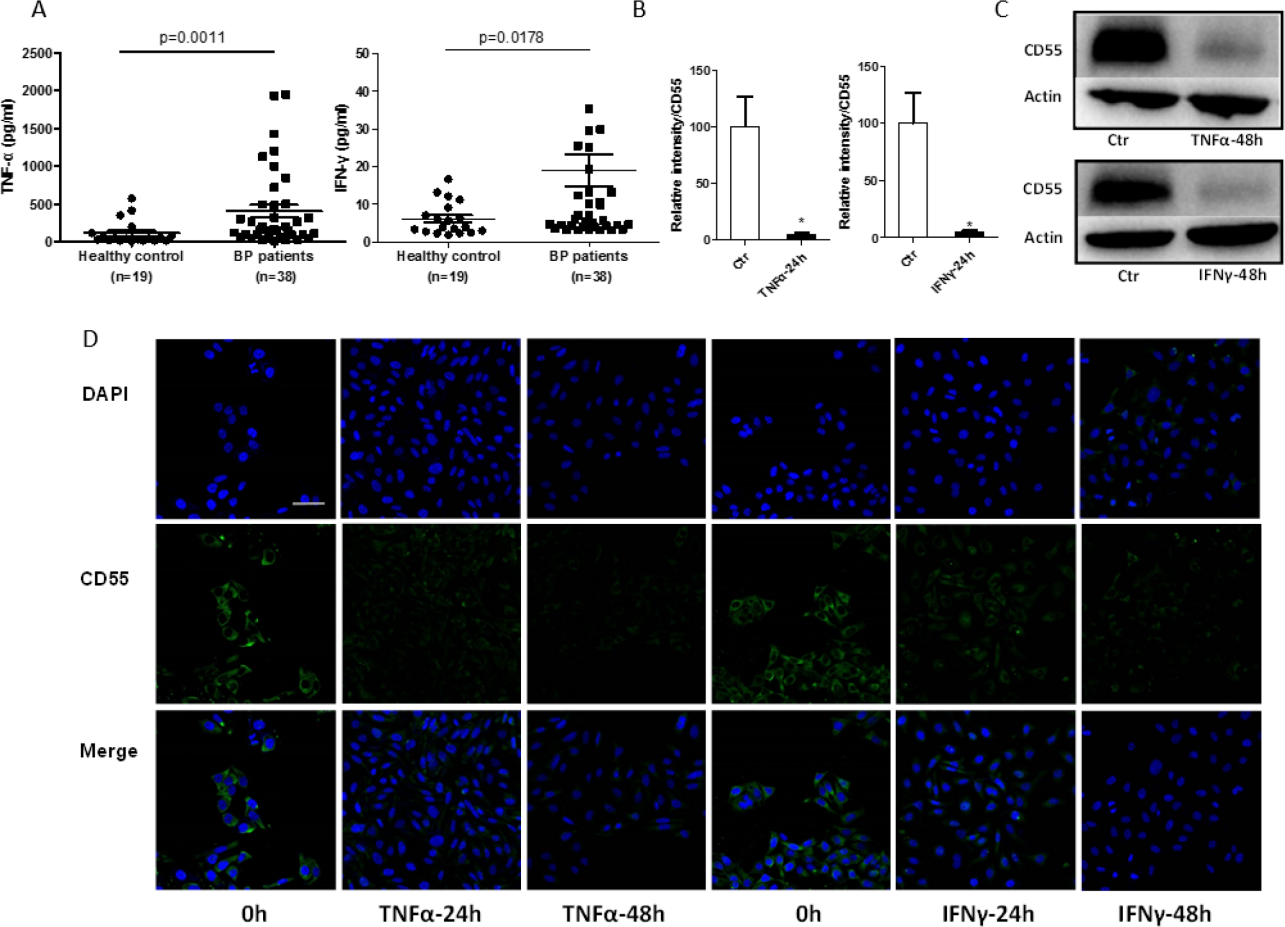
CD55 expression is downregulated by elevated TNF-α and IFN-γ levels in patients with bullous pemphigoid in HaCaT cells **(A)** An ELISA was used to determine TNF-α and IFN-γ levels in the sera of 38 patients with bullous pemphigoid and 19 healthy controls. **(B)** qRT-PCR, **(C)** western blotting, and **(D)** immunofluorescent staining were performed to measure CD55 expression after TNF-α (50 ng/ml) or IFN-γ (10 ng/ml) stimulation of HaCaT cells for 24 and 48 h, respectively. DAPI-stained nuclei can be seen in blue. Scale bar, 100 nm. Results represent means ± SEM from three independent experiments.

As previous studies have shown that cytokines play a major role in regulating the expression of proteins that inhibit the complement system [22], we measured levels of the cytokines TNF-α (TNF) and IFN-γ (IFNG) in the sera of 38 patients with bullous pemphigoid and 19 normal controls by enzyme-linked immunosorbent assay (ELISA). Serum TNF-α concentration among patients with bullous pemphigoid was 402.51 pg/ml, which is significantly higher than that among healthy controls (111.47 pg/ml). Consistent with this, serum IFN-γ concentration was also higher among the former (19.04 pg/ml) than the latter (6.14 pg/ml) (Figure 4A). To determine whether TNF-α and IFN-γ downregulate CD55 expression, we pretreated HaCaT cells with these cytokines at 50 and 10 ng/ml, respectively. As expected, quantitative reverse-transcription PCR (qRT-PCR) and western blotting revealed significantly decreased CD55 expression after TNF-α and IFN-γ stimulation for 24 and 48 h, respectively (Figure 4B and 4C). Immunofluorescent staining of CD55 on HaCaT cells treated with TNF-α and IFN-γ verified this result (Figure 4D). These findings indicate that serum levels of the cytokines TNF-α and IFN-γ are upregulated in bullous pemphigoid and that affects CD55 expression in keratinocytes.

### TNF-α and IFN-γ downregulate CD55 by activating ERK1/2 signaling pathway

**Figure 5:**
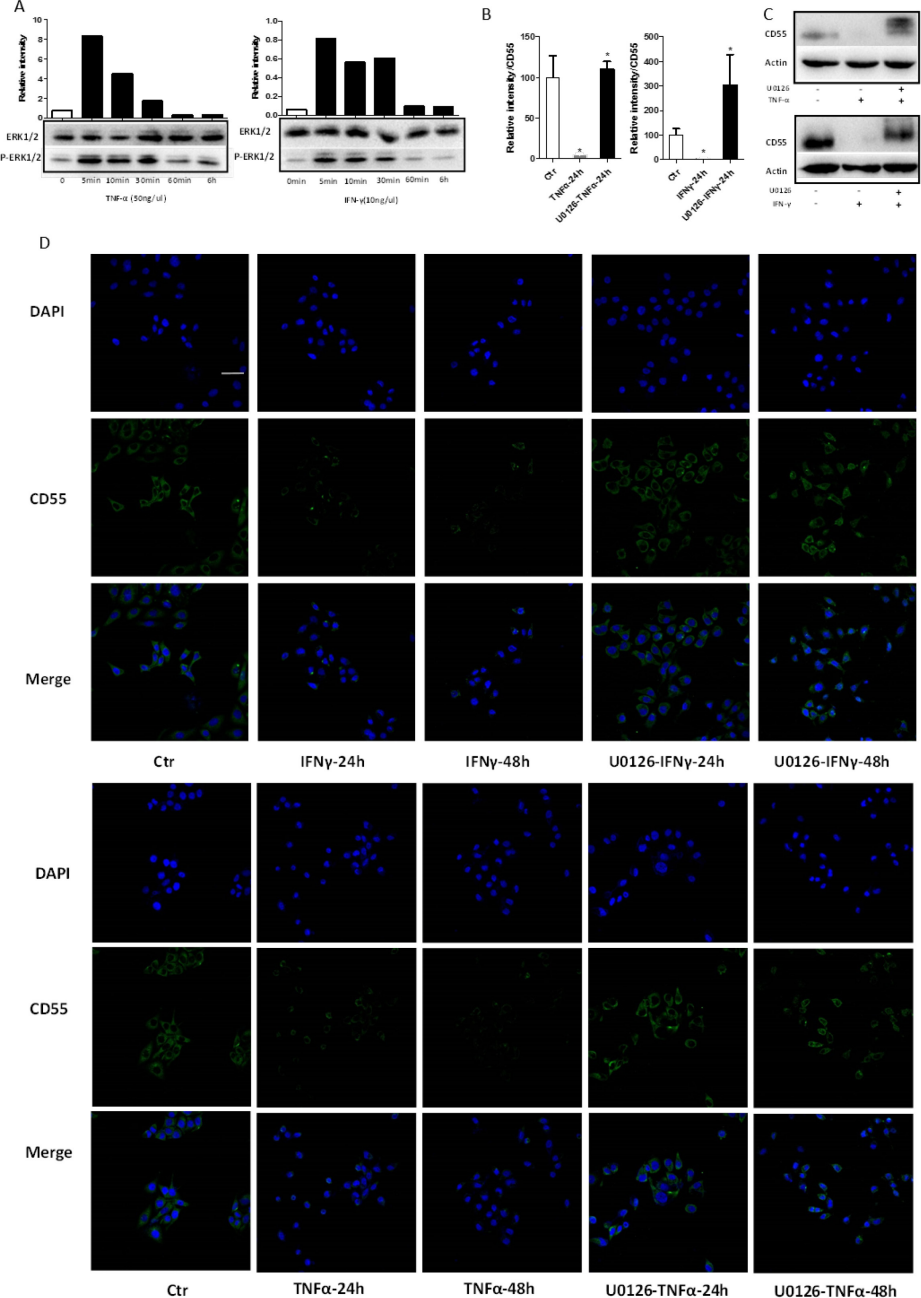
TNF-α and IFN-γ activate ERK1/2 signaling in HaCaT cells HaCaT cells were stimulated with TNF-α (50 ng/ml) or IFN-γ (10 ng/ml), and ERK1/2 and phospho-ERK1/2 levels were evaluated with the corresponding antibodies. **(A)** Levels of total and phospho-ERK1/2 were detected by western blotting. **(B)** qRT-PCR, **(C)** western blotting, and **(D)** immunofluorescent staining were performed to measure CD55 expression after stimulation of HaCaT cells with TNF-α (50 ng/ml) or IFN-γ (10 ng/ml) for 24 and 48 h, respectively, following pre-treatment with the ERK1/2 inhibitor U0126. DAPI-stained nuclei can be seen in blue. Scale bar, 100 nm. Results represent means ± SEM from three independent experiments.

As CD55 expression is associated with ERK-dependent signaling pathways, we examined phosphorylation of ERK1/2 (MAPK3/1) in TNF-α- and IFN-γ-treated HaCaT cells. Western blotting showed that both TNF-α (50 ng/ml) and IFN-γ (10 ng/ml) promoted tyrosine phosphorylation of ERK1/2 from 5 to 30 min after administration (Figure 5A). Thus, these cytokines can activate the ERK1/2 pathway in HaCaT cells.

To determine the mechanism by which TNF-α and IFN-γ decrease CD55 expression in HaCaT cells, the ERK1/2-specific antagonist U0126 was used to selectively inhibit ERK1/2 signaling. Pre-incubation of HaCaT cells with U0126 partially suppressed the effect of both TNF-α and IFN-γ (administered separately) on CD55 expression. qRT-PCR analysis showed that ERK1/2 inhibition resulted in significantly increased CD55 mRNA levels in cells treated with TNF-α or IFN-γ (Figure 5B). Moreover, western blotting confirmed the effects of partial ERK1/2 inhibition by U0126 pretreatment on TNF-α- or IFN-γ-mediated CD55 downregulation in HaCaT cells (Figure 5C). Finally, immunofluorescent staining revealed significantly increased CD55 signals as a result of U0126 pretreatment of cells stimulated with TNF-α or IFN-γ, consistent with our other findings (Figure 5D). In summary, an ERK1/2-specific antagonist (U0126) suppressed the effect of TNF-α and IFN-γ on CD55 expression at both the mRNA and protein levels.

## DISCUSSION

Bullous pemphigoid is a typical autoimmune disease and involves multiple mechanisms, including deposition of autoantibodies in the BMZ, complement activation, and the production of proinflammatory cytokines [23]. Activation of the complement system is critical in the development of this condition. It has been demonstrated that an experimental model of bullous pemphigoid cannot be induced in mice deficient of the key complement component C5. In addition, F(ab′)_2_ fragments generated from pathogenic anti-BP180 IgG fail to induce subepidermal blisters in C5-sufficient mice. Moreover, C5-deficient mice reconstituted with C5a become susceptible to experimental induction of the disease [8]. Wang et al. reported that the anti-BP180 Fabs B4 and 19, which lack the Fc portion necessary to activate the complement pathway, show therapeutic potential for bullous pemphigoid both *in vitro* and *in vivo*. These two fragments competitively inhibit the binding of bullous pemphigoid autoantibodies to the main BP180 NC16A epitope, blocking subsequent activation of complement components C1q and C3 [24, 25]. Together, these data suggest that dysregulation of complement activation is involved in bullous pemphigoid pathogenesis. However, the role of the factors directly regulate complement activation has not been fully clarified in this condition.

Regulation of complement inhibitors (CD55, CD35, CD59, and CD46) is important in autoantibody-mediated complement activation and is impaired in autoimmune diseases such as SLE, rheumatoid arthritis, and bullous pemphigoid [15]. Our recent study demonstrated that loss of the inhibitory function of the complement regulator CD46 may be involved in the pathogenesis of bullous pemphigoid. In the present study, we investigated the involvement of CD55, another critical complement regulator that accelerates the disassembly of preformed C3 and C5 convertases, in the development of this disease (Supplementary Figure 2). We found that CD55 levels were decreased in the lesional skin of patients but did not differ between the peripheral blood cells of either patients with the disease or in healthy controls. Moreover, CD55 depletion enhanced autoantibody-mediated complement activation as evidenced by elevated C3b deposition in HaCaT human keratinocytes, and administration of recombinant CD55 protected such cells from complement activation by pathogenic autoantibodies. In addition, elevated TNF-α and IFN-γ levels in patients with bullous pemphigoid downregulated CD55 via activation of ERK1/2 signaling. Our data show for the first time that compromised complement inhibition due to diminished CD55 levels may be implicated in the pathogenesis of bullous pemphigoid.

CD55 is a glycosyl phosphatidylinositol-anchored complement regulatory protein that binds to both classical and alternative C3 and C5 convertases, accelerating their decay and preventing C3b deposition and downstream assembly of the MAC complex in complement activation. CD55 is widely distributed among different cell types and its soluble form is found in tears, saliva, cerebrospinal fluid, urine, synovial fluid, and plasma. A previous study established that in patients with SLE, CD55 expression is decreased in lymphocytes, erythrocytes, and granulocytes, and negatively correlates with disease activity and complement activation [14, 19]. Consistent with this, Richaud-Patin et al. also demonstrated reduced CD55 expression in the membranes of erythrocytes from lupus patients with secondary AIHA, suggesting that aberrant CD55 levels play a role in the pathophysiology of certain autoimmune diseases [15, 18]. Moreover, using models of rheumatoid arthritis, a disorder in which complement activation and regulation has been implicated, Hoeck et al. found that mice lacking CD55 show reduced disease activity. This contrasts with other autoimmune conditions, in which CD55 deficiency seems to be an aggravating factor [26]. Here, we observed CD55 downregulation in the lesional skin of patients with bullous pemphigoid. Remarkably, this is the first time that abnormally decreased CD55 expression has been shown to accelerate the pathogenesis of this disease by promoting complement activation.

Bullous pemphigoid pathogenesis is accompanied by altered expression of numerous cytokines. Previous studies have shown that TNF-α and IFN-γ levels are increased in both the sera and blister fluids of patients with this condition, and that they correlate with disease activity [27]. Further work has shown that cytokines play a role in the regulation of complement inhibitory protein expression, and in particular, IFN-γ, TNF-α, IL-1β (IL1B), and TGF-β (TGFB) have been found to upregulate complement regulatory proteins in cultured tumor cells [28, 29]. In cultured human endothelial cells, Mason et al. showed that expression of CD55, but not CD46 or CD59, is increased by TNF-α and IFN-γ [30]. More recently, strong expression of CD55 on chondrocyte surfaces was demonstrated, and it was found that that CD55 is upregulated by stimulation with IL-1 in rheumatoid arthritis patients [15]. In our study, however, we recorded decreased CD55 expression after stimulating cultured HaCaT human keratinocytes with the cytokines TNF-α or IFN-γ, contrary to findings in other autoimmune diseases such as rheumatoid arthritis and SLE. To clarify the manner by which TNF-α and IFN-γ affect CD55 expression, the specific pathway involved was determined. Cui et al. reported that hepatitis B X-interacting protein is a novel regulator of CD55 in breast cancer cells, acting via ERK1/2/NF-κB (NFKB) signaling to contribute to the protection of cells from complement attack [31]. However, data concerning regulation of CD55 in autoimmune diseases are very limited. Our results demonstrated that CD55 was downregulated by TNF-α and IFN-γ via the ERK1/2 pathway, clarifying the involvement of abnormal complement activation in bullous pemphigoid pathogenesis.

Several studies have validated the therapeutic efficacy of recombinant sCR1, the short consensus repeat domain 1 of CD55, in various autoimmune and inflammatory disorders [32–34]. Here, we found that recombinant CD55 can inhibit C3b deposition in autoantibody-mediated complement activation, suggesting that complement-based therapies should be considered for the treatment of bullous pemphigoid. However, further studies will continue to investigate its therapeutic function in mouse models of this disease.

On the basis of the present results, it may be concluded that CD55 protein levels are significantly downregulated in the lesional skin of patients with bullous pemphigoid. Furthermore, such downregulation may be involved in the pathogenesis of this condition, for which replenishment of CD55 therefore constitutes a potential therapeutic strategy.

## MATERIALS AND METHODS

### Ethics statement

The study protocol was designed and approved by the medical research ethics review board of Xijing Hospital, the Fourth Military Medical University, Xi’an, China. Written informed consent was obtained from all participants prior to the study.

### Skin samples and human blood

Skin samples were obtained from 8 newly diagnosed patients with bullous pemphigoid and 6 healthy controls. The patients were diagnosed based on clinical symptoms and histological examination of skin samples, and had not received any systemic or local therapy for at least 2 weeks before lesional skin biopsies were obtained. Blood samples were obtained from 11 newly diagnosed patients with bullous pemphigoid (5 men and 6 women; age range: 45–81 years) and 11 healthy volunteers (6 men and 5 women; age range: 36–75 years) with no history of autoimmune diseases. Sera samples were obtained from 38 diagnosed patients with bullous pemphigoid and 19 healthy volunteers.

### Cell culture and TNF-α and IFN-γ treatment *in vitro*

HaCaT human keratinocytes were cultured in Dulbecco’s modified Eagle medium (DMEM) supplemented with 10% fetal bovine serum (Gibco, Invitrogen) in a humidified atmosphere containing 5% CO_2_ at 37°C. After 24 h of serum starvation in DMEM, the cells (60% confluency) were stimulated with TNF-α (50 ng/ml) or IFN-γ (10 ng/ml) (PeproTech Inc.) for 24 and 48 h, respectively. To inhibit ERK1/2 signaling, 10 mM U0126 (Sigma.) was added 1 h before stimulation with TNF-α or IFN-γ.

### qRT-PCR

Total RNA isolation and cDNA synthesis were performed as previously reported [35]. The quantitative PCR conditions were as follows: initial denaturation at 95°C for 10 s, followed by 40 cycles of denaturation at 95°C for 10 s, annealing at 54°C for 10 s, and extension at 72°C for 15 s. β-Actin was used as an internal control. Relative quantification was performed by the 2^–ΔΔCt^ method, and results are expressed as fold changes relative to the appropriate control. Each sample was analyzed in triplicate, and at least three independent experiments were performed. Results were considered significant when the difference in expression was at least 2-fold. The following CD55-specific primers were used: forward, TTTATTGTCCAGCACCACCA; reverse, TGCTCTCCAATCATGGTGAA.

### Western blotting

Cells or tissues were lysed with cell lysis solution as described previously [35]. The total protein concentration in lysates was measured with the bicinchoninic acid assay (Runde Biologicals Ltd, China); after which, equal amounts of protein were separated by SDS-polyacrylamide gel electrophoresis on 10% gels and transferred to a polyvinylidene difluoride membrane (Invitrogen, Carlsbad, CA, USA). The details of this protocol have been reported previously [35]. After blocking for 2 h with blocking buffer, the membrane was incubated overnight at 4°C with primary antibodies against CD55, β-actin, ERK1/2, and phospho-ERK1/2 (Abcam, Cambridge), before being incubated with horseradish peroxidase-conjugated goat anti-rabbit/mouse secondary antibodies (Dako, Glostrup) for 1 h at room temperature (20–25°C). Immunoreactivity was visualized by enhanced chemiluminescence. Protein expression levels were quantitated using the AlphaImager Gel Imaging System (Alpha Innotech).

### Immunofluorescence and confocal microscopy

As described above, HaCaT cells were seeded on glass bottom cell culture dishes (Nest Biotechnology) and treated with bullous pemphigoid auto-IgG and fresh serum from healthy controls containing complement components. The cells were rinsed twice with 0.01 mM PBS and fixed in freshly prepared 4% paraformaldehyde for 15 min, before being incubated with 0.3% Triton X-100 for 10 min at room temperature (20–25°C). Non-specific binding was blocked with 4% bovine serum albumin at 37°C for 40 min. After being rinsed three times with 0.01 mM PBS, the cells were incubated with a rabbit anti-human CD55 monoclonal antibody (1:200; Abcam) overnight at 4°C, followed by a fluorescein isothiocyanate (FITC)-labeled goat anti-rabbit IgG antibody for 1 h in the dark at 37°C. Nuclei were visualized by staining with 4′6-diamidino-2-phenylindole (DAPI). Tissue sections from patients with bullous pemphigoid and healthy controls were also treated with the anti-CD55 antibody, the FITC-conjugated goat anti-rabbit IgG antibody (Abcam) and DAPI, as described above. Samples were analyzed by confocal microscopy (FV-1000/ES; Olympus).

### Cell culture and CD55 interference

HaCaT human keratinocytes were cultured as previously described. After 24 h of serum starvation, cells grown to 60% confluence were transfected with *CD55* mRNA-targeting siRNA or negative control siRNA (100 nM; RibiBio) using Lipofectamine 3000 reagent (Invitrogen) according to the manufacturer’s instructions. Cells were harvested 48 h later.

### *In vitro* complement activation

This experiment was performed according to the method that we established in a previous study. Auto-IgG was purified from 15 ml of mixed serum from patients with bullous pemphigoid using HiTrap Protein G and a HiTrap *N*-hydroxy-succinimide-activated high-performance affinity column (Amersham Biosciences, Little Chalfont, UK) coated with the BP180 NC16A domain. HaCaT cells seeded on coverslips in 6-well plates were then incubated overnight with 1 μg/ml purified pathogenic IgG at 37°C, before being incubated for 2 h with 10 μg/ml recombinant CD55 protein and 1 ml of fresh serum from healthy controls containing complement components to initiate complement activation as a simulation of the bullous pemphigoid phenotype. C3b deposition at the DEJ and cell membrane was used as a measure of the degree of complement activation.

### Statistical analysis

All statistical analyses were performed using GraphPad Prism 5.0 (GraphPad Software, San Diego, CA, USA). P-values <0.05 were considered statistically significant. Data are expressed as means and standard errors of the means.

## SUPPLEMENTARY MATERIALS FIGURES



## Abbreviations

(DEJ): dermoepidermal junction
(BMZ): basement membrane zone
(MAC): membrane attack complex
(SLE): systemic lupus erythematosus
(AIHA): autoimmune hemolytic anemia
(ELISA): enzyme-linked immunosorbent assay
(DMEM): Dulbecco’s modified Eagle’s medium
(FITC): fluorescein isothiocyanate
(DAPI): 4’6-diamidino-2-phenylindole
(siRNA): short interfering RNA

## References

[1] Nousari HC, Anhalt GJ (1999). Pemphigus and bullous pemphigoid. Lancet.

[2] Culton DA, Liu Z, Diaz LA, Mackay IR, Rose NR (2014). Autoimmune Bullous Skin Diseases: Pemphigus and Pemphigoid. The Autoimmune Diseases, Fifth Edition.

[3] Di Zenzo G, Marazza G, Borradori L (2007). Bullous Pemphigoid: Physiopathology, Clinical Features and Management. Adv Dermatol.

[4] Schmidt E, della Torre R, Borradori L (2012). Clinical Features and Practical Diagnosis of Bullous Pemphigoid. Immunol Allergy Clin North Am.

[5] Liu Z, Giudice GJ, Swartz SJ, Fairley JA, Till GO, Troy JL, Diaz LA (1995). The role of complement in experimental bullous pemphigoid. J Clin Invest.

[6] Liu Z, Sui W, Zhao M, Li Z, Li N, Thresher R, Giudice GJ, Fairley JA, Sitaru C, Zillikens D, Ning G, Marinkovich MP, Diaz LA (2008). Subepidermal blistering induced by human autoantibodies to BP180 requires innate immune players in a humanized bullous pemphigoid mouse model. J Autoimmun.

[7] Lessey E, Li N, Diaz L, Liu Z (2008). Complement and cutaneous autoimmune blistering diseases. Immunol Res.

[8] Bajic G, Degn SE, Thiel S, Andersen GR (2015). Complement activation, regulation, and molecular basis for complement-related diseases. EMBO J.

[9] Noris M, Remuzzi G (2013). Overview of complement activation and regulation. Semin Nephrol.

[10] Sarma JV, Ward PA (2011). The complement system. Cell Tissue Res.

[11] Zipfel PF, Skerka C (2009). Complement regulators and inhibitory proteins. Nat Rev Immunol.

[12] Kim DD, Song WC (2006). Membrane complement regulatory proteins. Clin Immunol.

[13] Miwa T, Song WC (2001). Membrane complement regulatory proteins: insight from animal studies and relevance to human diseases. Int Immunopharmacol.

[14] Alegretti AP, Schneider L, Piccoli AK, Xavier RM (2012). The role of complement regulatory proteins in peripheral blood cells of patients with systemic lupus erythematosus: Review. Cell Immunol.

[15] Piccoli AK, Alegretti AP, Schneider L, Lora PS, Xavier RM (2011). Expression of complement regulatory proteins CD55, CD59, CD35, and CD46 in rheumatoid arthritis. Rev Bras Reumatol.

[16] Barclay AN, Brown MH, Law SKA, McKnight AJ, Tomlinson MG, van der Merwe PA (1997). The Leucocyte Antigen FactsBook, Second Edition.

[17] Ruiz-Argüelles A, Llorente L (2007). The role of complement regulatory proteins (CD55 and CD59) in the pathogenesis of autoimmune hemocytopenias. Autoimmun Rev.

[18] Richaud-Patin Y, Pérez-Romano B, Carrillo-Maravilla E, Rodriguez AB, Simon AJ, Cabiedes J, Jakez-Ocampo J, Llorente L, Ruiz-Argüelles A (2003). Deficiency of red cell bound CD55 and CD59 in patients with systemic lupus erythematosus. Immunol Lett.

[19] Alegretti AP, Mucenic T, Merzoni J, Faulhaber GA, Silla LM, Xavier RM (2010). Expression of CD55 and CD59 on peripheral blood cells from systemic lupus erythematosus (SLE) patients. Cell Immunol.

[20] García-Valladares I, Atisha-Fregoso Y, Richaud-Patin Y, Jakez-Ocampo J, Soto-Vega E, Elías-López D, Carrillo-Maravilla E, Cabiedes J, Ruiz-Argüelles A, Llorente L (2006). Diminished expression of complement regulatory proteins (CD55 and CD59) in lymphocytes from systemic lupus erythematosus patients with lymphopenia. Lupus.

[21] Soltys J, Halperin JA, Xuebin Q (2012). DAF/CD55 and Protectin/CD59 modulate adaptive immunity and disease outcome in experimental autoimmune myasthenia gravis. J Neuroimmunol.

[22] Qiao P, Dang E, Cao T, Fang H, Zhang J, Qiao H, Wang G (2017). Dysregulation of mCD46 and sCD46 contribute to the pathogenesis of bullous pemphigoid. Sci Rep.

[23] Song WC (2006). Complement regulatory proteins and autoimmunity. Autoimmunity.

[24] Sitaru C, Schmidt E, Petermann S, Munteanu LS, Bröcker EB, Zillikens D (2002). Autoantibodies to bullous pemphigoid antigen 180 induce dermal-epidermal separation in cryosections of human skin. J Invest Dermatol.

[25] Wang G, Ujiie H, Shibaki A, Nishie W, Tateishi Y, Kikuchi K, Li Q, McMillan JR, Morioka H, Sawamura D, Nakamura H, Shimizu H (2010). Blockade of autoantibody-initiated tissue damage by using recombinant fab antibody fragments against pathogenic autoantigen. Am J Pathol.

[26] Hoek RM, De Launay D, Kop EN, Yilmaz-Elis AS, Lin F, Reedquist KA, Verbeek JS, Medof ME, Tak PP, Hamann J (2010). Deletion of either CD55 or CD97 ameliorates arthritis in mouse models. Arthritis Rheum.

[27] Ludwig RJ, Schmidt E (2009). Cytokines in autoimmune bullous skin diseases. Epiphenomena or contribution to pathogenesis?. G Ital Dermatol Venereol.

[28] Schmitt CA, Schwaeble W, Wittig BM, Meyer Zum Büschenfelde KH, Dippold WG (1999). Expression and regulation by interferon-γ of the membrane-bound complement regulators CD46 (MCP), CD55 (DAF) and CD59 in gastrointestinal tumours. Eur J Cancer.

[29] Moutabarrik A, Nakanishi I, Namiki M, Hara T, Matsumoto M, Ishibashi M, Okuyama A, Zaid D, Seya T (1993). Cytokine-mediated regulation of the surface expression of complement regulatory proteins, CD46(MCP), CD55(DAF), and CD59 on human vascular endothelial cells. Lymphokine Cytokine Res.

[30] Mason JC, Yarwood H, Sugars K, Morgan BP, Davies KA, Haskard DO (1999). Induction of decay-accelerating factor by cytokines or the membrane-attack complex protects vascular endothelial cells against complement deposition. Blood.

[31] Cui W, Zhao Y, Shan C, Kong G, Hu N, Zhang Y, Zhang S, Zhang W, Zhang Y, Zhang X, Ye L (2012). HBXIP upregulates CD46, CD55 and CD59 through ERK1/2/NF-κB signaling to protect breast cancer cells from complement attack. FEBS Lett.

[32] Kremlitzka M, Polgár A, Fülöp L, Kiss E, Poór G, Erdei A (2013). Complement receptor type 1 (CR1, CD35) is a potent inhibitor of B-cell functions in rheumatoid arthritis patients. Int Immunol.

[33] Khera R, Das N (2009). Complement Receptor 1: disease associations and therapeutic implications. Mol Immunol.

[34] Ricklin D, Lambris JD (2007). Complement-targeted therapeutics. Nat Biotechnol.

[35] Shao S, Cao T, Jin L, Li B, Fang H, Zhang J, Zhang Y, Hu J, Wang G (2016). Increased Lipocalin-2 Contributes to the Pathogenesis of Psoriasis by Modulating Neutrophil Chemotaxis and Cytokine Secretion. J Invest Dermatol.

