# Supplementary material for: Decreased expression levels of complement regulator CD55 contribute to the development of bullous pemphigoid
Source: Oncotarget. 2017 Sep 23;9(85):35517–27. doi: 10.18632/oncotarget.21216 (PMC6238980; doi:10.18632/oncotarget.21216)
Supplement: Supplementary file 1 [file oncotarget-09-35517-s001.pdf]

Decreased expression levels of complement regulator CD55 contribute to the development of bullous pemphigoid

SUPPLEMENTARY MATERIALS

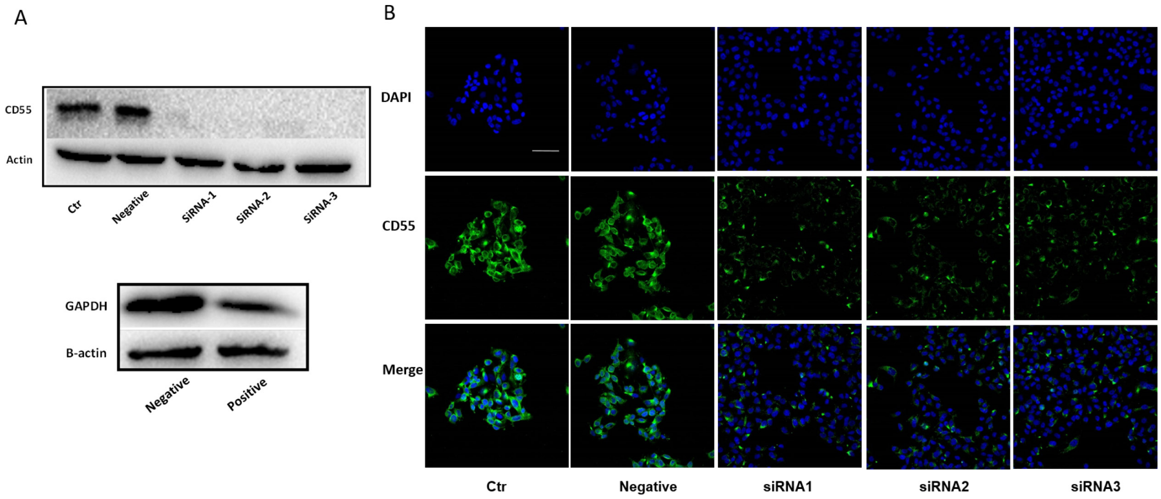

Supplementary Figure 1: Short interference RNA targeting CD55 was transfected into keratinocytes to detect the expression of CD55 protein. The effectiveness of anti-CD55 siRNA and transfection system relative to that of the negative control was determined by western blot **(A)** and immunofluorescent staining **(B)** after 48 h of transfection. Scale bar, 100 nm.

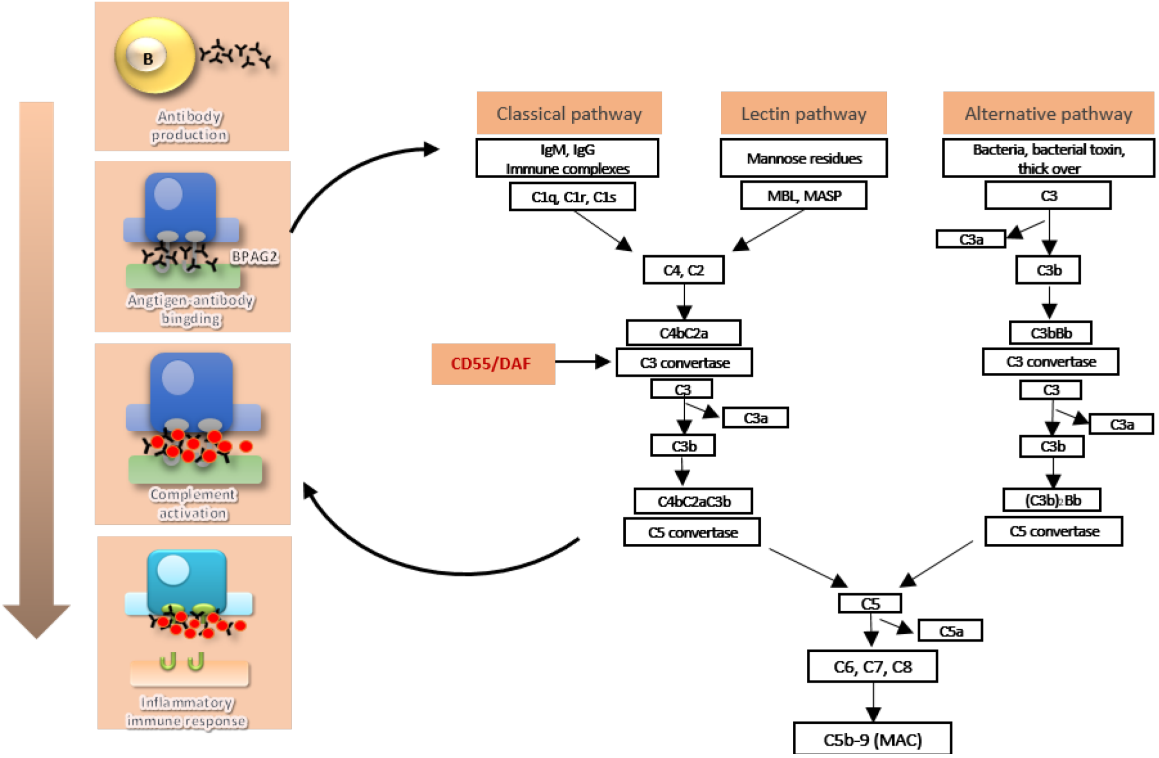

Supplementary Figure 2: Schematic diagram of the pathogenic role of CD55 in the development of bullous pemphigoid.
